# Bacterial Communities in the Heads and Gasters of 26 Ant Taxa

**DOI:** 10.3390/insects17090910

**Published:** 2026-08-31

**Authors:** Zixin Pei, Jianlong Zhang, Haoyan Bai, Manyu Li, Liangliang Zhang, Hong He

**Affiliations:** 1 College of Forestry, Northwest A&F University, Yangling 712100, China; pzx1790072608@gmail.com (Z.P.); jlzhang@nwafu.edu.cn (J.Z.); limyokk@outlook.com (M.L.); zhang2l@nwafu.edu.cn (L.Z.); 2 College of Plant Protection, Northwest A&F University, Yangling 712100, China; hybai2002@nwafu.edu.cn

**Keywords:** Formicidae, bacterial community, high-throughput sequencing, host phylogeny, honeydew-associated ants

## Abstract

Insect-associated bacterial communities can vary with host diet, phylogeny, and body region. In this study, we used 16S ribosomal RNA (rRNA) gene amplicon sequencing and a host phylogenetic tree constructed from mitochondrial genes to analyze bacterial communities in whole-head and whole-gaster samples from 26 ant taxa, including 23 honeydew-associated taxa and three dietary comparators. Pseudomonadota was the predominant phylum across most sampled taxa and samples. Chao1 richness and Shannon diversity were significantly higher in head samples than in paired gaster samples, whereas the overall difference in bacterial community structure between body regions was significant but small. No uniform bacterial profile was shared by all 23 honeydew-associated taxa. Gaster bacterial community structure showed significant congruence with host phylogeny, whereas no significant congruence was detected in head communities. Certain bacterial taxa exhibited patterns associated with specific hosts or body regions, including *Candidatus* Blochmannia in Camponotini gasters, and *Fructilactobacillus* in the heads of *Oecophylla smaragdina*. Overall, bacterial communities differed markedly among host taxa and showed more limited variation between body regions, providing a comparative basis for further studies of ant-associated bacteria.

## Simple Summary

Bacteria associated with ants may vary with host identity, diet, and body region. Honeydew is carbohydrate-rich but comparatively limited in nitrogenous nutrients, and many ants use it as one component of a broader diet. In this study, we compared bacterial communities in whole-head and whole-gaster samples from 26 ant taxa, including 23 honeydew-associated taxa and three dietary comparators. Bacterial composition varied markedly among the sampled taxa and showed more limited differences between body regions, and no single bacterial community pattern was shared by all 23 honeydew-associated taxa. Gaster bacterial community patterns also corresponded significantly with the evolutionary relationships among the sampled ants, whereas this correspondence was not detected in head communities. Certain bacterial taxa exhibited distribution patterns associated with specific hosts or body regions, including *Candidatus* Blochmannia in the gasters of Camponotini ants and *Fructilactobacillus* in the heads of *Oecophylla smaragdina*. These findings show that ant-associated bacterial communities differ among host taxa and body regions and provide a comparative basis for future research on the ecological roles of these bacteria.

## 1. Introduction

Ants (Hymenoptera: Formicidae) are ecologically successful social insects with diverse feeding habits and extensive interactions with plants and other arthropods [1]. Flowering plants provide ants with diverse food and habitat resources, and ant–plant interactions have evolved repeatedly across ant groups [1,2]. Many ants exploit liquid carbohydrate resources, including extrafloral nectar and honeydew excreted by sap-feeding hemipterans [3,4]. Associations between ants and honeydew-producing hemipterans are widespread and often take the form of food-for-protection mutualisms, in which hemipterans provide carbohydrate-rich honeydew and attending ants may protect the hemipterans from predators and parasitoids [4,5]. However, the strength and outcome of these interactions are context dependent [4,5,6]. Honeydew feeding is an important component of the foraging ecology of many ant groups. Among ant subfamilies, Formicinae, Myrmicinae, and Dolichoderinae include many taxa that interact with honeydew-producing hemipterans [4]. *Tetraponera rufonigra* has been observed attending *Aphis craccivora* and collecting its honeydew [7].

Insects harbor diverse bacterial associates that can contribute to host nutrition, metabolism, and development [8,9]. Some bacteria establish persistent symbiotic relationships with their hosts, whereas others may be acquired transiently from food or the surrounding environment [10]. The composition of bacterial communities in insects is shaped by multiple factors, including diet, host phylogeny, habitat, developmental stage, and so on [10,11,12]. Bacterial communities can also differ among anatomical regions within the host body. The head frequently contacts food and the external environment during activities such as foraging and trophallaxis. It also contains the infrabuccal pocket, a preoral structure that filters and stores solid particles from ingested material [13]. By contrast, the gaster contains the crop, proventriculus, midgut, and hindgut, which are involved in food storage, microbial filtration, digestion, absorption, and nutrient processing [14]. This functional differentiation of tissues and organs may contribute to spatial variations in microbial composition.

Host diet and phylogeny are among the factors associated with bacterial community composition in ants [8,15,16]. For example, Russell et al. [16] reported that Rhizobiales gut symbionts are closely associated with herbivorous ant groups, particularly arboreal ants that rely heavily on plant-derived exudates. Honeydew is a sugar-rich resource but is relatively poor in nitrogenous compounds, including amino acids. Consequently, ants that use honeydew may therefore obtain nitrogen from complementary food sources, while bacterial symbionts contribute to nitrogen recycling or essential amino acid biosynthesis in some ant groups [4,17,18,19,20].

Previous studies have examined ant-associated bacterial communities in relation to diet, phylogeny, nesting mode, and digestive tract compartment [8,14,15,16]. However, many studies have focused on individual host taxa, restricted taxonomic groups, or specific digestive-tract compartments. Consequently, comparative information on bacterial communities across taxonomically diverse ants that include honeydew in their diets remains limited. In this study, “honeydew-associated” refers to taxa with published records of honeydew consumption or trophobiotic interactions with honeydew-producing hemipterans. These taxa may differ in their degree of reliance on honeydew, ranging from highly dependent trophobiotic taxa to omnivorous taxa that include honeydew as one component of a broader diet. We used 16S ribosomal RNA (rRNA) gene amplicon sequencing to compare bacterial communities in whole-head and whole-gaster samples from 26 ant taxa, including 23 honeydew-associated taxa and three dietary comparators. We aimed to characterize variation in bacterial community composition among the sampled taxa, colonies, and body regions and to assess whether bacterial community structure was congruent with host phylogenetic configuration. This study provides comparative information on the diversity and distribution of ant-associated bacterial communities across different host taxa and body regions.

## 2. Materials and Methods

### 2.1. Sample Collection and Identification

From May to November 2025, foraging workers were collected from multiple localities in China, including Yangling District, Ningshan County, and Zhouzhi County in Shaanxi Province; Xishuangbanna in Yunnan Province; and Guangzhou in Guangdong Province. For each taxon, workers were collected from at least three nests. Each nest was sampled only once and was treated as an independent biological replicate. Sampled nests were separated by a minimum distance of 20 m, although the actual distances were often greater. Workers from different sampled nests showed aggressive responses when brought into contact and were therefore considered to represent different colonies. Ant specimens were identified by the authors based on external morphological characters, published taxonomic descriptions, distribution records, and the online resources Ants-China and AntWiki [21,22]. Representative voucher specimens of the sampled taxa were retained in the laboratory, College of Forestry, Northwest A&F University, Yangling, China.

Of the 26 sampled taxa, 25 were identified to species level, whereas one *Crematogaster* taxon could be identified only to genus level and is therefore reported as *Crematogaster* sp. throughout the manuscript. Based on published records of honeydew consumption or trophobiotic interactions with honeydew-producing hemipterans, 23 taxa were assigned to the honeydew-associated category. The remaining three taxa were included as dietary comparators: the seed-harvesting ant *Messor aciculatus* and the predatory ants *Brachyponera chinensis* and *Odontomachus monticola*. Evidence supporting the dietary classification of each taxon is summarized in Table S1 [7,14,23,24,25,26,27,28,29,30,31,32,33]. Sample information is summarized in Table 1, while detailed colony-level environmental metadata are provided in Table S2. Air temperature and relative humidity were measured at the time of collection using a thermo-hygrometer, whereas geographic coordinates and elevation were recorded using a global positioning system device.

### 2.2. Sample Preparation

On a clean bench, the ant samples were surface-sterilized with 75% ethanol for 3 min, rinsed three times with sterile water, and dried with sterile filter paper. Dissections were performed on sterilized glass slides under a stereomicroscope (Olympus Corporation, Tokyo, Japan). The heads and gasters of the ants were carefully separated using sterile forceps, transferred into individual 1.5 mL sterile centrifuge tubes, and stored at −80 °C. To minimize the risk of cross-contamination, the forceps were flame-sterilized after each sample, and a new sterile glass slide was utilized for each dissection.

Both head and gaster samples were prepared for each ant taxon, with at least three colony replicates per taxon. To obtain sufficient material for DNA extraction, workers were divided into three body-size classes based on their body lengths. For each colony replicate, heads or gasters from 10 large workers (≥5.5 mm), 20 medium-sized workers (3.5 to <5.5 mm), or 40 small workers (<3.5 mm) were pooled. A total of 186 samples were obtained from the 26 ant taxa, comprising 93 head samples (H) and 93 gaster samples (G).

### 2.3. DNA Extraction, PCR Amplification, and High-Throughput Sequencing

Total genomic deoxyribonucleic acid (DNA) was extracted from the ant samples using the E.Z.N.A.^®^ DNA Kit (Omega Bio-tek, Norcross, GA, USA) according to the manufacturer’s instructions. DNA quality was assessed by 1% agarose gel electrophoresis, and DNA concentration and purity were determined using a NanoDrop 2000 spectrophotometer (Thermo Fisher Scientific, Waltham, MA, USA).

Using the extracted DNA as a template, the hypervariable V3–V4 region of the bacterial 16S rRNA gene was amplified by polymerase chain reaction (PCR) with primer pairs 338F (5′-ACTCCTACGGGAGGCAGCAG-3′) and 806R (5′-GGACTACHVGGGTWTCTA AT-3′) [34], which incorporated barcode sequences. The PCR reaction mixture (20 μL) contained 4 μL of 5× TransStart FastPfu buffer, 2 μL of 2.5 mM dNTPs, 0.8 μL of each primer (5 μM), 0.4 μL of TransStart FastPfu DNA Polymerase (TransGen Biotech Co., Ltd., Beijing, China), 1 μL of template DNA, and ddH_2_O to reach the final volume. PCR amplification cycling conditions were as follows: initial denaturation at 95 °C for 3 min; followed by 26 cycles of denaturation at 95 °C for 30 s, annealing at 55 °C for 30 s, and extension at 72 °C for 30 s; and a final extension at 72 °C for 10 min, ending at 4 °C. The PCR products were excised from a 2% agarose gel and purified using a DNA Gel Extraction Kit (YuHua, Shanghai, China). A PCR no-template control, with sterile water replacing template DNA, was included. No visible amplification product was detected by agarose gel electrophoresis (Figure S1), and the control was not included in the 16S rRNA sequencing run. The purified products were then quantified using a Qubit 4.0 Fluorometer (Thermo Fisher Scientific, Waltham, MA, USA).

DNA extraction and PCR amplification for all 186 samples were each performed in a single batch under identical experimental conditions. Amplicon libraries corresponding to the samples included in this study were pooled in equimolar amounts and sequenced together in a single run on an Illumina NextSeq 2000 platform (Illumina, San Diego, CA, USA) at Majorbio Bio-Pharm Technology Co., Ltd. (Shanghai, China), using a 2 × 300 bp paired-end configuration. The expected length of the V3–V4 amplicon was approximately 468 bp. The raw sequences have been submitted to the National Center for Biotechnology Information (NCBI) Sequence Read Archive (SRA) database under BioProject PRJNA1481559.

### 2.4. Bioinformatic and Statistical Analysis

Raw paired-end reads were quality-filtered using fastp (version 0.19.6) [35]. Bases at the 3′ end were trimmed when the mean quality score within a 50 bp sliding window was below 20. Reads shorter than 50 bp after trimming and reads containing ambiguous bases were discarded. The filtered reads were merged using FLASH (version 1.2.11) [36], with a minimum overlap of 10 bp and a maximum mismatch ratio of 0.2 in the overlapping region. No barcode mismatches and a maximum of two primer mismatches were allowed. The resulting merged sequences were processed using DADA2 [37] in QIIME 2 (version 2020.2) to infer amplicon sequence variants (ASVs) and remove chimeric sequences, using the parameters applied by the sequencing provider. Taxonomic assignment was performed using the classify-sklearn Naive Bayes classifier against the SILVA 138.2 bacterial 16S rRNA reference database (silva138.2/16s_bacteria), with a confidence threshold of 0.7. Sequences assigned to chloroplasts or mitochondria were removed before downstream analyses. A total of 9,962,331 denoised sequences were retained across the 186 samples, with 33,187–94,439 sequences per sample. For downstream diversity analyses, the ASV table was rarefied to 27,849 sequences per sample. The resulting rarefied dataset contained 16,768 ASVs across the 186 samples.

Alpha diversity was assessed using the Chao1 richness index and the Shannon diversity index at the ASV level, calculated with Mothur (version 1.30.1) [38]. Differences in Chao1 richness and Shannon diversity between paired head and gaster samples were evaluated using two-sided Wilcoxon signed-rank tests, with samples from the same colony treated as paired observations (*n* = 93 colony pairs). *p* values were calculated using the normal approximation with continuity correction, as implemented in the ‘stats’ package of R (version 4.5.2). Taxon-level alpha-diversity values were summarized descriptively. Beta diversity was assessed using principal coordinate analysis (PCoA) based on Bray–Curtis dissimilarities. Group differences were tested using permutational multivariate analysis of variance (PERMANOVA) with 9999 permutations, and homogeneity of multivariate dispersion was evaluated using permutational analysis of multivariate dispersions (PERMDISP). For comparisons between paired head and gaster samples, permutations were restricted within colonies. Lingoes correction was applied to the PCoA when required to address negative eigenvalues. Analyses were performed using the ‘vegan’ package in R (version 4.5.2). To assess the influence of dominant intracellular symbionts, beta-diversity analyses were repeated after removing ASVs assigned to *Wolbachia* alone or to both *Wolbachia* and *Candidatus* Blochmannia. Relative abundances were renormalized after removal. Because no ASVs remained in Cj2G after both genera were removed, the Cj2 pair was excluded and all three datasets were analyzed using the same 92 colony pairs without adding pseudocounts.

To identify dominant bacterial taxa that differed between head and gaster samples and determine which of the 26 ant taxa showed higher relative abundances of these bacteria, analyses were restricted to the 20 most abundant taxa in the genus-level dataset across all 186 samples. Paired head and gaster samples were compared using two-sided Wilcoxon signed-rank tests. Differences among ant taxa within each body region were assessed using Kruskal–Wallis tests. For bacterial taxa that differed significantly between body regions, one-sided focal-versus-rest Wilcoxon rank-sum tests were then performed within the corresponding body region. *p*-values were adjusted for multiple testing using the Benjamini–Hochberg (BH) method, and rank-biserial correlations were calculated as effect sizes. For the heatmap visualization, asterisks are shown only for comparisons with BH-adjusted *p* < 0.05, rank-biserial *r* ≥ 0.50, and median relative abundance ≥ 2%; heatmap values represent Z-scores of taxon-level mean relative abundance.

### 2.5. Phylogenetic Analysis of the Host Ants

To construct the host phylogenetic tree, three mitochondrial genes—cytochrome c oxidase subunit I (*COI*), cytochrome b (*Cytb*), and the small subunit ribosomal RNA (12S rRNA)—were amplified by PCR. The PCR reaction was performed in a 25 μL volume containing 4 μL of template DNA, 1 μL of each of the forward and reverse primers (10 μM), 12.5 μL of 2× Accurate Taq Master Mix (Accurate Biotechnology (Hunan) Co., Ltd., Changsha, China), and 6.5 μL of sterile, nuclease-free water. The primer pairs used were as follows: LCO1490 (5′-GGTCAACAAATCATAAAGATATT GG-3′) and HCO2198 (5′-TAAACTTCAGGGTGACCAAAAAATCA-3′) for *COI* [39]; CB1 (5′-TATGTACTACCATGAGGACAAATATC-3′) and CB2 (5′-ATTACACCTCCTAATTTATTAGGAAT-3′) for *Cytb* [40]; and 12Sma (5′-CTGGGATTAGATACCCTGTTAT-3′) and 12Smb (5′-CAGAGAGTGACGGGCGATTTGT-3′) for 12S rRNA [41]. The PCR cycling conditions were as follows: initial denaturation at 94 °C for 30 s, followed by 35 cycles of denaturation at 94 °C for 30 s, annealing at 52.4 °C (*COI*), 48 °C (*Cytb*), or 52 °C (12S rRNA) for 40 s, and extension at 72 °C for 40 s, with a final extension at 72 °C for 2 min. The PCR products were verified by 1% agarose gel electrophoresis and subsequently submitted to Sangon Biotech (Shanghai, China) for sequencing.

To ensure data quality and check for the presence of premature stop codons, the protein-coding *COI* and *Cytb* were translated into amino acid sequences using MEGA (version 11.0) [42]. Multiple sequence alignment was performed using MAFFT (version 7.0) [43]. The aligned sequences were then trimmed to remove poorly aligned regions using GBlocks (version 0.91b) and concatenated using PhyloSuite (version 2) [44,45]. The best-fit nucleotide substitution models were identified using ModelFinder (version 2.2.0) [46]. A maximum likelihood (ML) phylogenetic tree was constructed using IQ-TREE (version 2.2.0) [47], with branch support assessed by 1000 ultrafast bootstrap replicates [48]. All gene sequences used to construct the phylogenetic tree were uploaded to the NCBI GenBank database, numbered as PZ571225-PZ571250 (*COI*), PZ576150-PZ576175 (*Cytb*), and PZ574104-PZ574129 (12S rRNA). Outgroup mitochondrial gene sequences were obtained from NCBI GenBank, including *Vespula germanica* (OM735807.1) and *Vespa mandarinia* (PX583370.1).

### 2.6. Procrustes Analysis of Host Phylogeny and Bacterial Community Structure

To assess congruence between host phylogeny and bacterial community structure, genus-level relative abundances were averaged across colonies within each ant taxon separately for head and gaster samples. Patristic distances among the 26 ant taxa were calculated from the mitochondrial phylogenetic tree constructed as described in Section 2.5, whereas Bray–Curtis dissimilarities were calculated from the corresponding taxon-level bacterial profiles. Each distance matrix was subjected to principal coordinate analysis with Cailliez correction [49]. Symmetric Procrustes analysis was performed using the first five axes from each ordination, and statistical significance was assessed using PROTEST with 9999 permutations [50]. The analysis was conducted for the complete dataset and repeated for the symbiont-removal datasets described in Section 2.4. *p*-values were adjusted for multiple testing using the Benjamini–Hochberg method.

## 3. Results

### 3.1. Bacterial Community Composition Across the 26 Sampled Ant Taxa

#### 3.1.1. Phylum Level Composition

**Figure 1:**
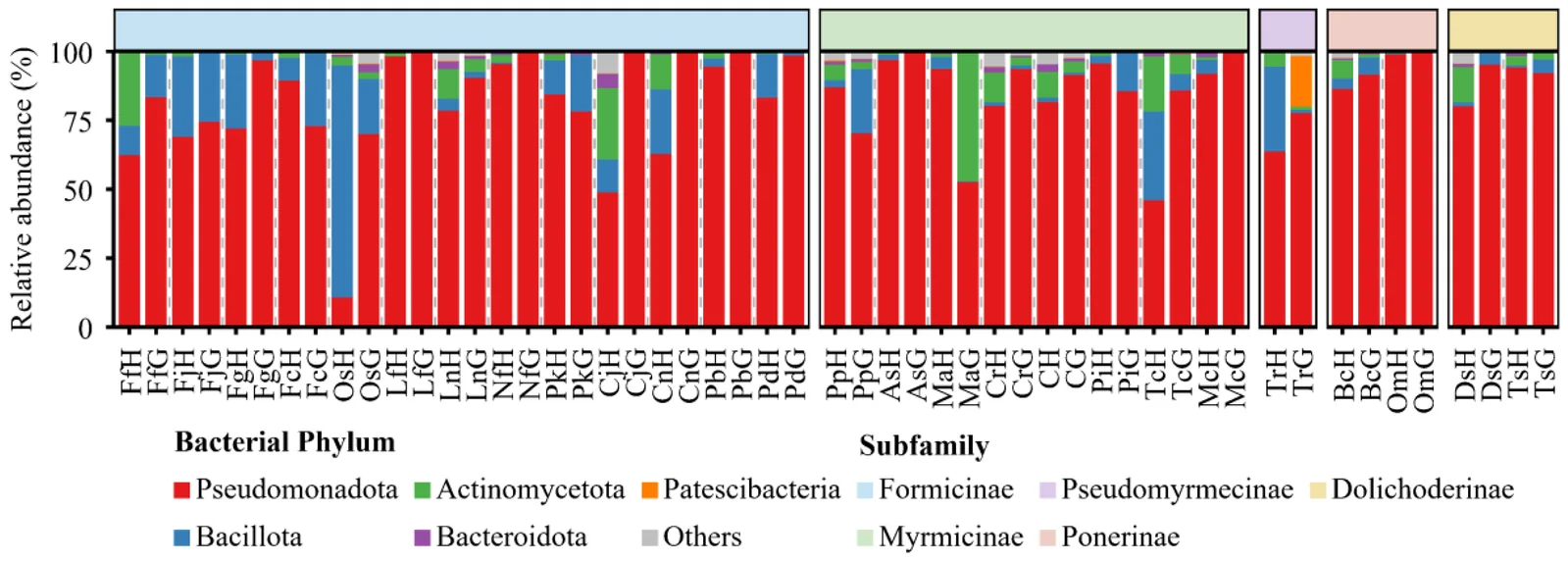
Bacterial community composition at the phylum level in the heads and gasters of 26 ant taxa (note: H and G indicate head and gaster samples, respectively).

At the phylum level, the bacterial communities were mainly composed of Pseudomonadota, Bacillota, and Actinomycetota (Figure 1 and Figure S2). Pseudomonadota was the predominant phylum in most taxa and samples. Bacillota showed a relatively high average abundance in certain ants belonging to the subfamilies Formicinae, Myrmicinae, and Pseudomyrmecinae. It was the single dominant phylum in the heads of *Oecophylla smaragdina* (OsH, 84.20 ± 4.44%). Actinomycetota occurred at a relatively high abundance in the gasters of *Messor aciculatus* (MaG, 47.05 ± 1.95%). Bacteroidota and the remaining phyla generally occurred at low relative abundances, although Patescibacteria was more prominent in some gaster samples of *Tetraponera rufonigra* (TrG). Replicate colonies of most taxa showed broadly similar phylum-level profiles within the same body region, although the relative proportions of Pseudomonadota and Bacillota varied among colonies in some taxa (Figure S2).

#### 3.1.2. Genus Level Composition

**Figure 2:**
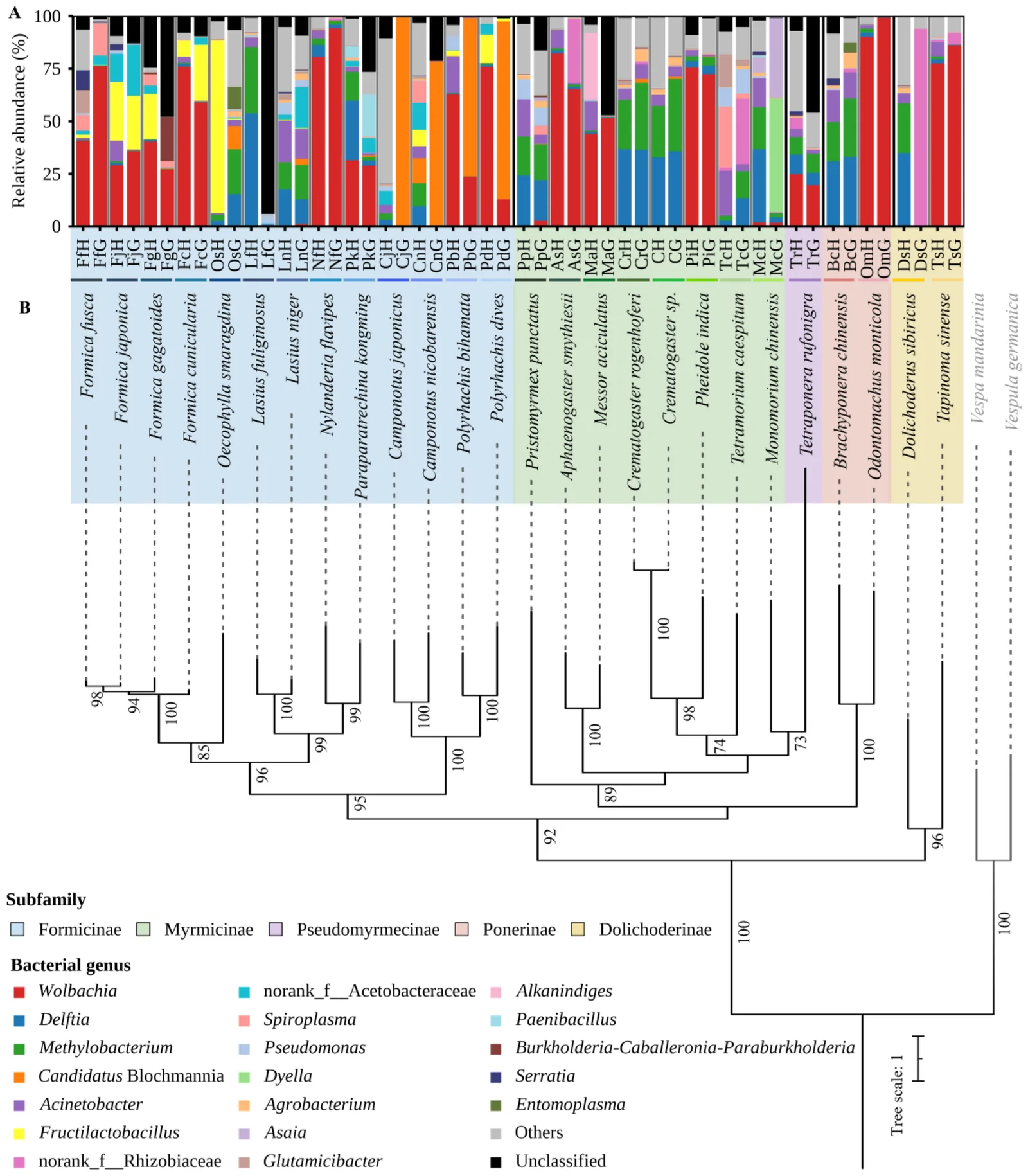
Genus-level bacterial community composition and host phylogeny of the 26 ant taxa. (**A**) Bacterial community composition at the genus level in the heads and gasters of 26 ant taxa. (**B**) Host phylogenetic tree of the study taxa based on concatenated mitochondrial genes (note: the numbers on the branches represent the bootstrap support rate; outgroups are shown in gray).

At the genus level, bacterial community composition varied markedly among host taxa and between body regions (Figure 2A,B and Figure S3). *Wolbachia* was the most abundant genus overall and dominated the bacterial communities of several ant taxa across multiple subfamilies. It reached an average of 99.32 ± 0.08% in the gasters of *Odontomachus monticola* (OmG), and was also the predominant genus in *Nylanderia flavipes* (Nf), *Messor aciculatus* (Ma), *Aphaenogaster smythiesii* (As), *Pheidole indica* (Pi), and *Tapinoma sinense* (Ts), as well as in several *Formica* samples (Ff, Fj, Fg and Fc) and the heads of *Polyrhachis* species (PbH and PdH). *Delftia* and *Methylobacterium* were most prevalent in the subfamily Myrmicinae. Specifically, they were the dominant genera in two *Crematogaster* taxa (C and Cr), *Pristomyrmex punctatus* (Pp), as well as the heads of *Monomorium chinensis* (McH). These genera were also relatively abundant in *Brachyponera chinensis* (Bc), *Lasius niger* (Ln), the heads of *Oecophylla smaragdina* (OsH), the heads of *L. fuliginosus* (LfH), and the heads of *Dolichoderus sibiricus* (DsH). An unclassified member from the family Acetobacteraceae (norank_f__Acetobacteraceae) was detected in both head and gaster samples and reached relatively high abundances in some samples from several Formicinae taxa, including *Lasius niger*, *Formica*, *Camponotus*, and *Polyrhachis*.

Several bacterial taxa showed distinct distributions among host taxa and body regions (Figure 2A,B and Figure S3). *Candidatus* Blochmannia was dominant in the gasters of Camponotini ants, including *Camponotus* and *Polyrhachis* species. *Fructilactobacillus* was almost exclusively present in certain genera of Formicinae. It was relatively abundant in some *Formica* samples (Fc, Fj, and Fg), and emerged as the overwhelmingly dominant genus in the heads of *Oecophylla smaragdina* (82.12 ± 5.72%). It was also relatively abundant in the heads of *Polyrhachis* species and *Camponotus nicobarensis* (CnH). An unclassified member of Rhizobiaceae (norank_f__Rhizobiaceae) was dominant in the gasters of *Dolichoderus sibiricus* (93.92 ± 2.98%).

At the individual colony level, replicate colonies of most taxa showed broadly similar dominant genera within the same body region, whereas other taxa exhibited substantial variation among colonies (Figure S3). For example, the gasters of *Aphaenogaster smythiesii* were dominated by *Wolbachia* in two colonies, whereas norank_f__Rhizobiaceae predominated in the gaster of another colony, reaching 91.21%. The composition of the dominant bacterial genera in the gasters of *Tetramorium caespitum* also varied among different colonies, being dominated by relatively high abundances of *Delftia* and *Methylobacterium*, *Pseudomonas*, or norank_f__Rhizobiaceae.

### 3.2. Bacterial Community Diversity and Structure

#### 3.2.1. Alpha Diversity

**Figure 3:**
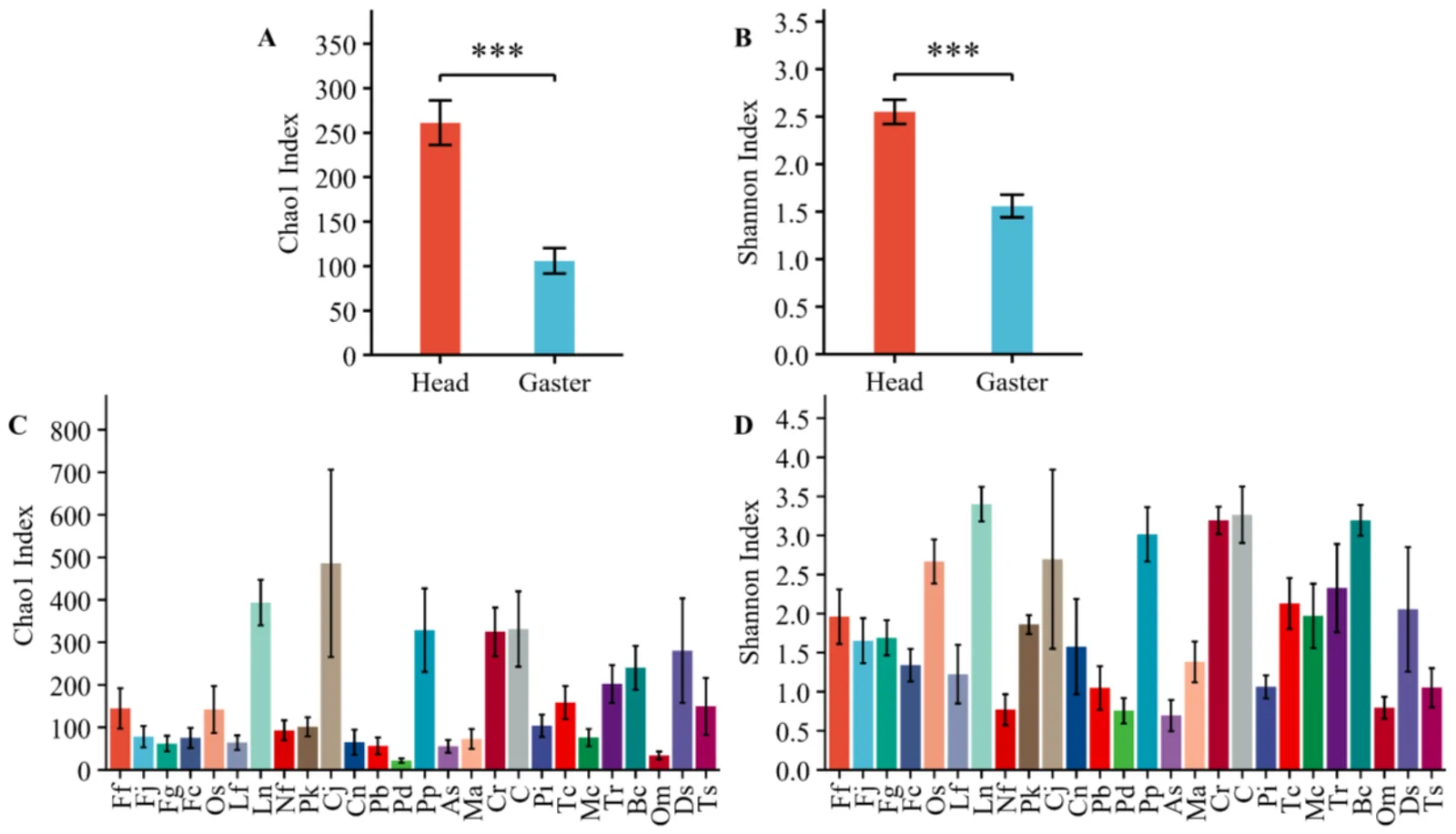
Alpha diversity indices of bacterial communities in the heads and gasters of 26 ant taxa at the amplicon sequence variant (ASV) level. (**A**) Chao1 index of ant heads and gasters. (**B**) Shannon index of ant heads and gasters. (**C**) Chao1 index across the 26 ant taxa. (**D**) Shannon index across the 26 ant taxa (note: differences between paired head and gaster samples were evaluated using two-sided Wilcoxon signed-rank tests, with colony identity defining the paired observations (*n* = 93 colony pairs); *p* ≤ 0.001 is marked with ***; error bars represent standard errors; taxon abbreviations are defined in Table 1).

Both Chao1 richness and Shannon diversity were significantly higher in head samples than in paired gaster samples (Chao1: V = 4081, *p* < 0.001; Shannon: V = 4001, *p* < 0.001; *n* = 93 colony pairs; Figure 3A,B; Table S3). At the taxon level, both indices also varied markedly among the 26 ant taxa (Figure 3C,D; Table S4). Mean Chao1 richness was highest in *Camponotus japonicus* (485.63 ± 220.32) and *Lasius niger* (393.64 ± 53.56), and lowest in *Polyrhachis dives* (21.72 ± 5.13). Mean Shannon diversity was highest in *L. niger* (3.40 ± 0.22) and lowest in *Aphaenogaster smythiesii* (0.70 ± 0.20).

#### 3.2.2. Beta Diversity

**Figure 4:**
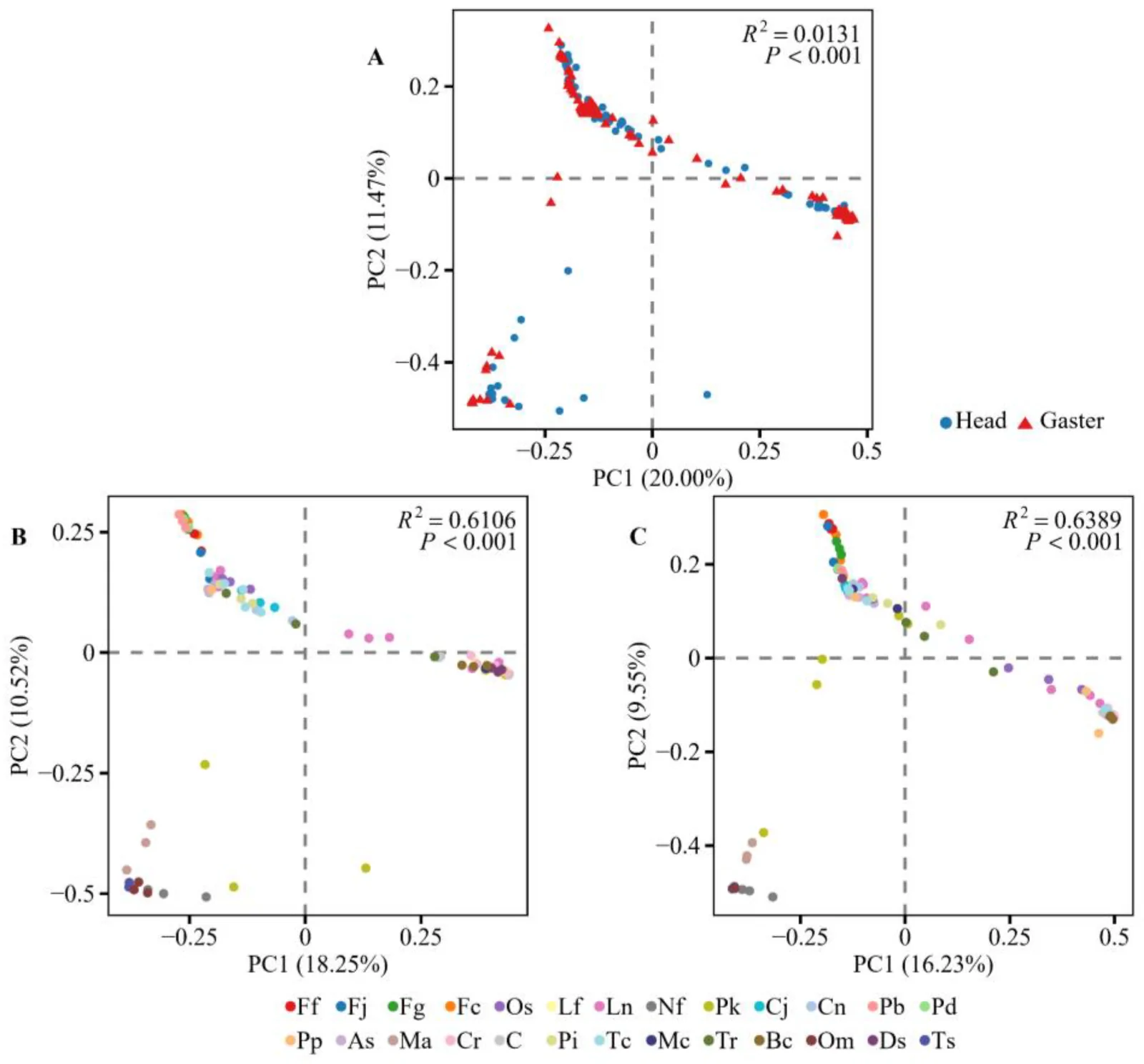
Principal coordinate analysis of bacterial communities based on Bray–Curtis dissimilarities. (**A**) Paired head and gaster samples. (**B**) Head samples among the 26 ant taxa. (**C**) Gaster samples among the 26 ant taxa.

PCoA showed substantial overlap between paired head and gaster samples (Figure 4A). Nevertheless, paired PERMANOVA detected a statistically significant but limited difference in bacterial community composition between the two body regions (pseudo-*F* = 2.438, *R*^2^ = 0.0131, *p* < 0.001; *n* = 93 colony pairs). Multivariate dispersion also differed significantly between head and gaster samples (PERMDISP: *F* = 5.144, *p* < 0.001). When the two body regions were analyzed separately, bacterial community composition differed markedly among the 26 ant taxa in both head samples (pseudo-*F* = 4.203, *R*^2^ = 0.6106, *p* < 0.001; Figure 4B) and gaster samples (pseudo-*F* = 4.742, *R*^2^ = 0.6389, *p* < 0.001; Figure 4C). PERMDISP was also significant for both datasets (head: *F* = 4.197, *p* < 0.001; gaster: *F* = 2.923, *p* < 0.001).

After removal of *Wolbachia* and after removal of both *Wolbachia* and *Candidatus* Blochmannia, differences between body regions remained significant but small (*R*^2^ = 0.0199 and 0.0182, respectively; both *p* < 0.001; Table S5). Differences among the 26 ant taxa also remained significant in head samples (*R*^2^ = 0.5603 and 0.5607, respectively) and gaster samples (*R*^2^ = 0.6332 and 0.5817, respectively; both *p* < 0.001). PERMDISP remained significant for the head–gaster comparison after removal of *Wolbachia* (*p* = 0.0034) and after removal of both genera (*p* = 0.0140). Among-taxon dispersion also differed significantly in gaster samples under both removal treatments (both *p* = 0.0004), but not in head samples (*p* = 0.0561 and 0.0773, respectively; Table S5).

#### 3.2.3. Congruence Between Host Phylogeny and Bacterial Community Structure

Procrustes analysis showed different patterns of congruence between host phylogeny and bacterial community structure in the two body regions (Table S6). No significant congruence with host phylogeny was detected for head bacterial communities (Procrustes *r* = 0.336, BH-adjusted *p* = 0.5799), whereas gaster bacterial communities showed significant congruence (Procrustes *r* = 0.505, BH-adjusted *p* = 0.0082). After the removal of *Wolbachia*, congruence remained significant in gaster samples (Procrustes *r* = 0.611, BH-adjusted *p* < 0.001), whereas no significant congruence was detected in head samples. After removal of both *Wolbachia* and *Candidatus* Blochmannia, neither head nor gaster communities showed significant congruence with host phylogeny after BH correction (head: Procrustes *r* = 0.420, BH-adjusted *p* = 0.1400; gaster: Procrustes *r* = 0.458, BH-adjusted *p* = 0.0826).

### 3.3. Differences in Dominant Bacterial Genera

**Figure 5:**
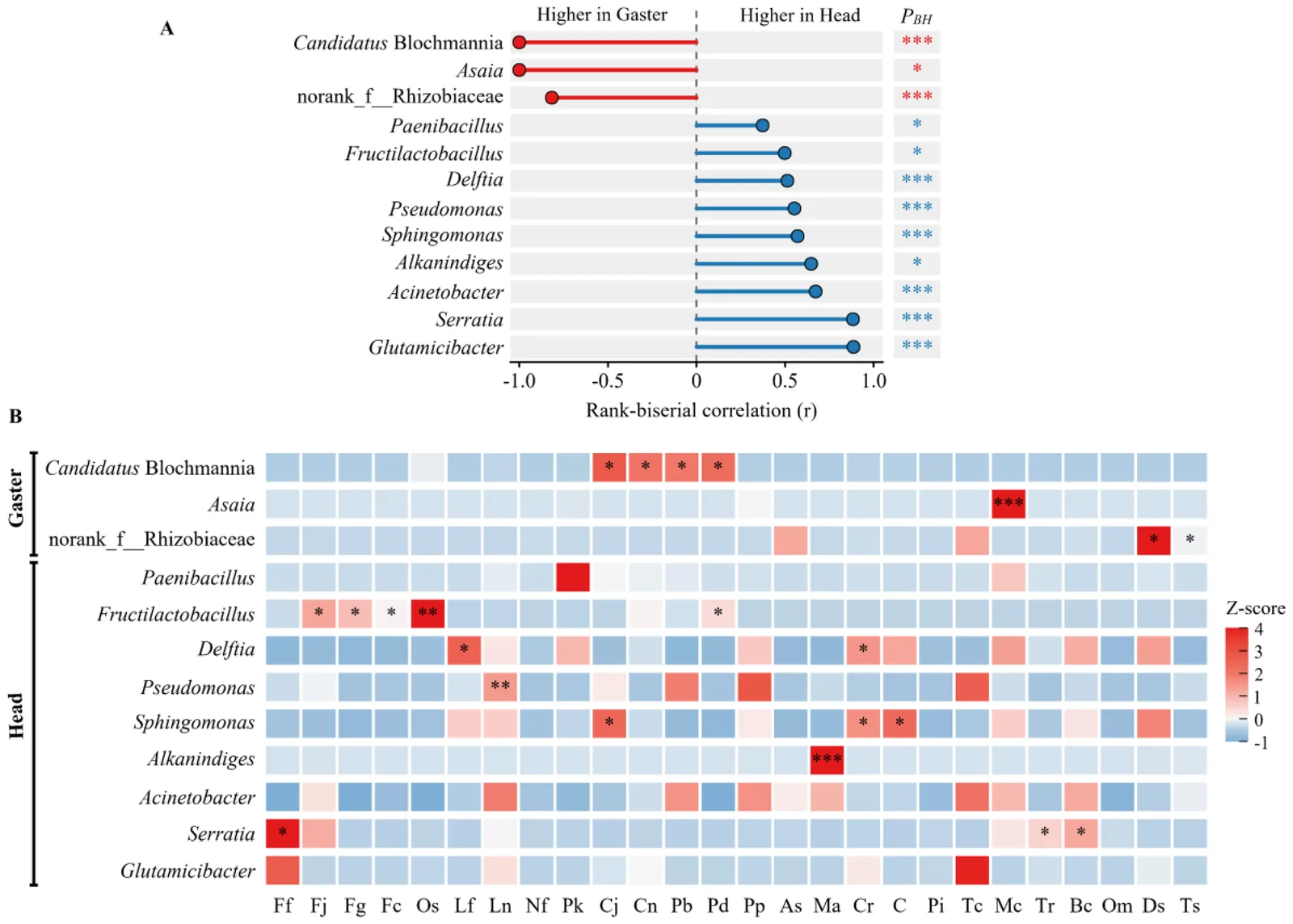
Differences in dominant bacterial taxa between body regions and among the 26 ant taxa. (**A**) Rank-biserial correlations for paired comparisons between head and gaster samples. (**B**) Distribution of bacterial genera that differed between head and gaster samples among the 26 ant taxa; colors indicate Z-scores calculated within each genus (note: in (**B**), asterisks are shown only for comparisons meeting the effect size and abundance criteria defined in the methods; * *p*_BH_ < 0.05, ** *p*_BH_ < 0.01, and *** *p*_BH_ < 0.001).

Among the 20 dominant bacterial taxa, 12 differed significantly between paired head and gaster samples after BH correction (Figure 5A; Table S7). *Candidatus* Blochmannia, norank_f__Rhizobiaceae, and *Asaia* showed higher relative abundance ranks in gaster samples, whereas *Acinetobacter*, *Glutamicibacter*, *Serratia*, *Sphingomonas*, *Delftia*, *Pseudomonas*, *Fructilactobacillus*, *Paenibacillus*, and *Alkanindiges* showed higher ranks in head samples.

These bacterial taxa also showed distinct distributions among the 26 ant taxa (Figure 5B; Table S8). *Candidatus* Blochmannia was particularly abundant in the gasters of *Camponotus* and *Polyrhachis* species, whereas norank_f__Rhizobiaceae was most prominent in the gaster of *Dolichoderus sibiricus*. In head samples, *Fructilactobacillus* was particularly abundant in *Oecophylla smaragdina* and also showed higher relative abundances in *Formica japonica*, *F. gagatoides*, *F. cunicularia*, and *Polyrhachis dives*. *Asaia* was particularly abundant in the gaster of *Monomorium chinensis*, whereas *Alkanindiges* was particularly abundant in the head of *Messor aciculatus*.

## 4. Discussion

Bacteria associated with ants include both persistent symbionts and taxa that may be acquired transiently from food or the surrounding environment [10,51]. Across the 26 ant taxa examined in this study, Pseudomonadota, Bacillota, and Actinomycetota dominated the bacterial communities at the phylum level, whereas composition at the genus level varied markedly among host taxa and body regions. Although 23 sampled taxa were associated with honeydew use, they did not share a uniform genus-level bacterial profile. Instead, similarities were restricted to subsets of host taxa. Previous studies have identified host diet and taxonomy as important correlates of insect-associated bacterial communities [52,53,54]. In this study, host phylogenetic configuration was significantly congruent with bacterial community structure in the gaster, but not in the head. This gaster congruence remained significant after the removal of *Wolbachia* alone, but was no longer significant after the additional removal of *Candidatus* Blochmannia following BH correction. The phylogenetic congruence observed in gaster communities may therefore be partly influenced by the high relative abundance and concentrated distribution of *Candidatus* Blochmannia in Camponotini ants.

Beyond variation among the sampled taxa, bacterial communities also differed between the head and gaster. Chao1 richness and Shannon diversity were higher in head samples, whereas the difference in overall community structure between body regions was statistically significant but small and remained small after removal of *Wolbachia* alone or both *Wolbachia* and *Candidatus* Blochmannia. Among the bacterial taxa differing between body regions, *Candidatus Blochmannia* and norank_f__Rhizobiaceae showed higher relative abundances in gaster samples, whereas *Pseudomonas* and *Delftia* were more abundant in head samples. These patterns may partly reflect differences in environmental exposure and in the anatomical structures contained within the two body regions [55,56]. The head frequently contacts food, nest materials, and external surfaces during foraging and trophallaxis and contains the infrabuccal pocket (IBP), which filters particles from ingested liquids [13]. The gaster mainly contains the digestive tract, such as the crop, proventriculus, midgut, and hindgut, which perform major functions in food storage, digestion, absorption, and microbial filtration [57]. These anatomical differences provide a plausible context for the observed patterns; however, because whole-head and whole-gaster samples were analyzed, the differences cannot be attributed to specific structures.

Among the bacterial taxa that differed between body regions, *Candidatus* Blochmannia showed the clearest association with both body region and host lineage, consistent with its role as a nutritional symbiont. It predominated in the gasters of *Camponotus* and *Polyrhachis* species, consistent with its known association with Camponotini ants. Previous genomic and experimental studies have shown that *Blochmannia* contributes to nitrogen recycling and essential amino acid biosynthesis, thereby improving host nutrition [58,59]. These established functions provide a plausible nutritional context for its high relative abundance in the gasters. Furthermore, Wernegreen et al. [60] showed that *Blochmannia* is phylogenetically related to endosymbionts of sap-feeding hemipterans and proposed that ancient trophobiotic interactions with these insects may have provided an ecological opportunity for the ancestor of Camponotini to acquire the ancestral *Blochmannia* symbiont in association with honeydew feeding. However, such clear host-lineage and body-region associations were not observed across all symbionts. In this study, *Wolbachia* was widely distributed across five ant subfamilies and was dominant in multiple host species. Previous studies have reported that *Wolbachia* is widespread in ants and other social insects, with infection patterns associated with host taxonomy, biogeography, and social behavior [61]. Horizontal transmission may also influence the distribution of *Wolbachia* across different host taxa in ants [62]. Thus, the high abundance of *Wolbachia* in some hosts may contribute to among-taxon differences in bacterial composition but does not represent a feature specific to honeydew-associated ants. Marked differences among taxa remained after removal of these dominant symbionts.

Some bacterial taxa detected in this study have also been reported from carbohydrate-rich environments or sugar-feeding insects. *Fructilactobacillus* showed higher relative abundances in head samples and was particularly abundant in *Oecophylla smaragdina*. Higher abundances were also observed in *Formica japonica*, *F. gagatoides*, *F. cunicularia*, and *Polyrhachis dives*. All of these host taxa have previously been reported to feed on honeydew or engage in trophobiotic interactions with honeydew-producing hemipterans [14,23,24]. Previous studies have shown that lactic acid bacteria (LAB) are prevalent in the infrabuccal pockets and crops of ants that prefer honeydew [14], and *Fructilactobacillus sanfranciscensis* was reported as the predominant lactic acid bacterium isolated from the infrabuccal pockets and crops of *F*. *cunicularia* [63]. Members of the Lactobacillales have also been documented in diverse ant genera, including *Formica*, *Lasius*, *Cephalotes*, and *Tapinoma* [11,14,64]. An unclassified member of the family Acetobacteraceae (norank_f__Acetobacteraceae) was detected in both body regions and reached relatively high abundances in some samples from several Formicine taxa, including *Lasius niger*, *Formica*, *Camponotus*, and *Polyrhachis*. Members of Acetobacteraceae are commonly associated with carbohydrate-rich environments and have frequently been reported from sugar-feeding insects [65], including *Camponotus* [66] and *Lasius* ants [67]. These observations are consistent with bacterial occurrence in ants using carbohydrate-rich food resources, but they do not demonstrate a honeydew-specific association because most taxa in the present dataset were honeydew-associated and honeydew intake was not quantified.

Other bacterial taxa may be more closely associated with environmental exposure. *Delftia* and *Methylobacterium* occurred across both ground-associated and arboreal ant taxa. *Delftia* has been reported from soils and plant rhizospheres, whereas *Methylobacterium* is commonly associated with plants [68,69]. Their occurrence in multiple ant taxa is therefore consistent with environmental acquisition, although the present data cannot distinguish transient acquisition from persistent host association.

Several limitations should be noted. Because the number of workers pooled per replicate was adjusted according to body size, differences in pooling number should be considered when interpreting taxon-level alpha-diversity patterns. The PCR no-template control produced no visible amplicon by agarose gel electrophoresis and was not included in the sequencing run; therefore, background DNA below the detection threshold of the gel assay was not independently characterized. Finally, 16S rRNA gene amplicon sequencing describes bacterial composition but cannot establish bacterial function, viability, or stable colonization. Future studies with more balanced dietary sampling, bacterial cultivation, and metagenomic or metatranscriptomic approaches will be needed to clarify the origins and functions of key bacterial taxa [20].

## 5. Conclusions

This study shows that bacterial communities associated with ants vary among host taxa and between body regions and that honeydew-associated taxa do not exhibit a shared bacterial community pattern. These patterns suggest that host identity, body region, and phylogenetic relationships contribute to bacterial community composition among ants. The stronger congruence between gaster bacterial communities and host phylogeny suggests that gaster-associated bacterial communities are more closely linked to host evolutionary relationships, whereas head bacterial communities may be more influenced by environmental exposure. Together, these findings highlight the importance of considering host characteristics and differences between body regions when investigating insect–microbe interactions.

## Figures and Tables

**Table 1:** Sample information record.

Subfamily	Ant Taxon	Taxon ID	Collection Locality	No. of Colonies	No. of Samples
Formicinae	*Formica fusca*	Ff	Yangling District, Shaanxi Province	3	6
	*Formica japonica*	Fj	Ningshan County, Shaanxi Province	3	6
	*Formica gagatoides*	Fg	Ningshan County, Shaanxi Province	3	6
	*Formica cunicularia*	Fc	Yangling District, Shaanxi Province	3	6
	*Oecophylla smaragdina*	Os	Xishuangbanna, Yunnan Province	3	6
	*Lasius fuliginosus*	Lf	Ningshan County, Shaanxi Province	3	6
	*Lasius niger*	Ln	Yangling District, Shaanxi Province	11	22
	*Nylanderia flavipes*	Nf	Zhouzhi County, Shaanxi Province	3	6
	*Paraparatrechina kongming*	Pk	Yangling District and Ningshan County, Shaanxi Province	5	10
Formicinae	*Camponotus japonicus*	Cj	Yangling District, Shaanxi Province	3	6
	*Camponotus nicobarensis*	Cn	Guangzhou, Guangdong Province	3	6
	*Polyrhachis dives*	Pd	Xishuangbanna, Yunnan Province	3	6
	*Polyrhachis bihamata*	Pb	Xishuangbanna, Yunnan Province	3	6
Myrmicinae	*Pristomyrmex punctatus*	Pp	Yangling District and Ningshan County, Shaanxi Province	3	6
	*Aphaenogaster smythiesii*	As	Ningshan County, Shaanxi Province	3	6
	*Messor aciculatus*	Ma	Ningshan County, Shaanxi Province	3	6
	*Crematogaster rogenhoferi*	Cr	Yangling District, Shaanxi Province	6	12
	*Crematogaster* sp.	C	Yangling District, Shaanxi Province	3	6
	*Pheidole indica*	Pi	Yangling District, Shaanxi Province	3	6
	*Tetramorium caespitum*	Tc	Yangling District, Shaanxi Province	5	10
	*Monomorium chinensis*	Mc	Yangling District, Shaanxi Province	3	6
Pseudomyrmecinae	*Tetraponera rufonigra*	Tr	Xishuangbanna, Yunnan Province	3	6
Ponerinae	*Brachyponera chinensis*	Bc	Ningshan County, Shaanxi Province	3	6
	*Odontomachus monticola*	Om	Ningshan County, Shaanxi Province	3	6
Dolichoderinae	*Tapinoma sinense*	Ts	Ningshan County, Shaanxi Province	3	6
	*Dolichoderus sibiricus*	Ds	Yangling District, Shaanxi Province	3	6
Total	26 ant taxa	-	-	93	186

Note: Each colony contributed one head sample and one gaster sample. Taxon IDs are used throughout the figures and supplementary materials. Detailed colony-level sampling information is provided in Table S2.

## Data Availability

The data supporting this study can be obtained from NCBI. The GenBank numbers are PZ571225-PZ571250 (*COI*), PZ576150-PZ576175 (*Cytb*), and PZ574104-PZ574129 (12S rRNA). The BioProject number is PRJNA1481559.

## Supplementary Materials

The following supporting information can be downloaded at https://www.mdpi.com/article/10.3390/insects17090910/s1. Figure S1. Agarose gel electrophoresis of bacterial 16S rRNA gene PCR products from the biological samples and the PCR no-template control; Figure S2. Relative abundance of dominant bacterial phyla in individual head and gaster samples of different ant taxa; Figure S3. Relative abundance of dominant bacterial genera in individual head and gaster samples of different ant taxa; Figure S4. Principal coordinate analysis of bacterial community structure after removal of *Wolbachia* and *Candidatus* Blochmannia; Table S1. Dietary classification and supporting evidence for the 26 sampled ant taxa [7,14,23,24,25,26,27,28,29,30,31,32,33]; Table S2. Colony-level sampling metadata and field observations; Table S3. Statistical comparisons of alpha diversity between paired head and gaster; Table S4. Alpha-diversity statistics for pooled samples from the 26 sampled ant taxa; Table S5. PERMANOVA and PERMDISP results for bacterial community structure before and after removal of *Wolbachia* and *Candidatus* Blochmannia; Table S6. Congruence between host phylogenetic configuration and genus-level bacterial community structure assessed by symmetric Procrustes analysis and PROTEST; Table S7. Paired comparisons of dominant bacterial genera between head and gaster samples; Table S8. Ant taxa highlighted in Figure 5B for bacterial taxa differing between body regions.
