# Supplementary material for: Bacterial Communities in the Heads and Gasters of 26 Ant Taxa
Source: Insects. 2026 Aug 31;17(9):910. doi: 10.3390/insects17090910 (PMC13607509; doi:10.3390/insects17090910)
Supplement: Supplementary file 1 [file insects-17-00910-s001.zip › Supplementary Figures.pdf]

Sumpplementary material

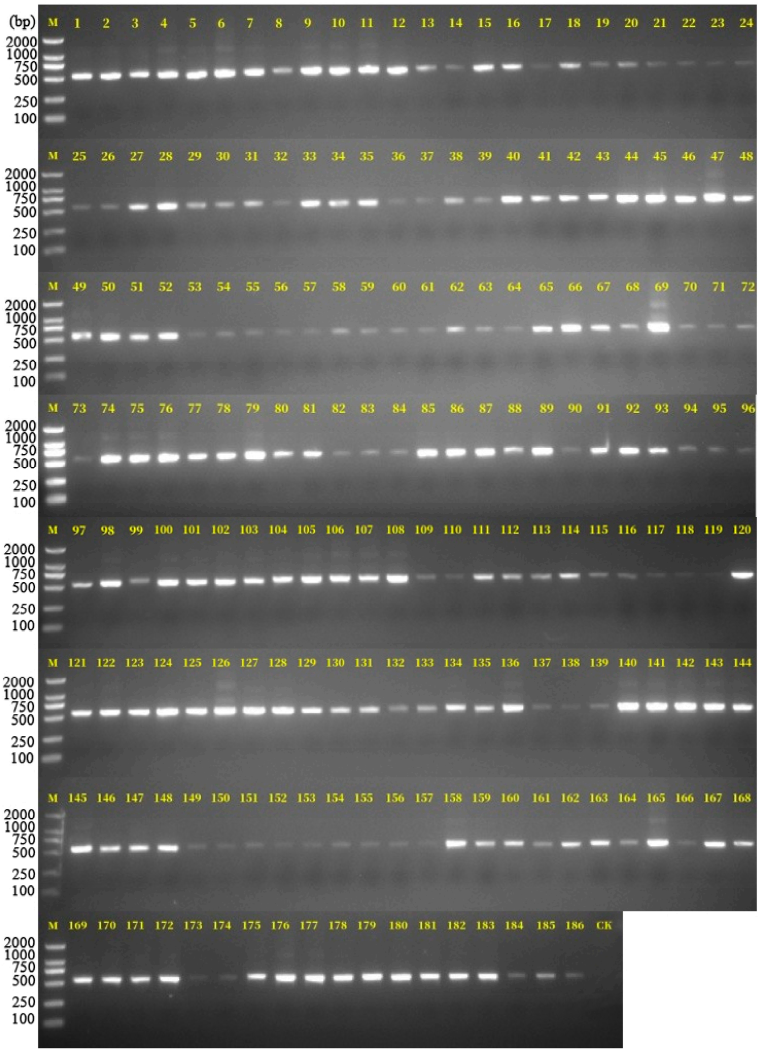

Figure S1. Agarose gel electrophoresis of bacterial *16S rRNA* gene PCR products from the biological samples and the PCR no-template control.

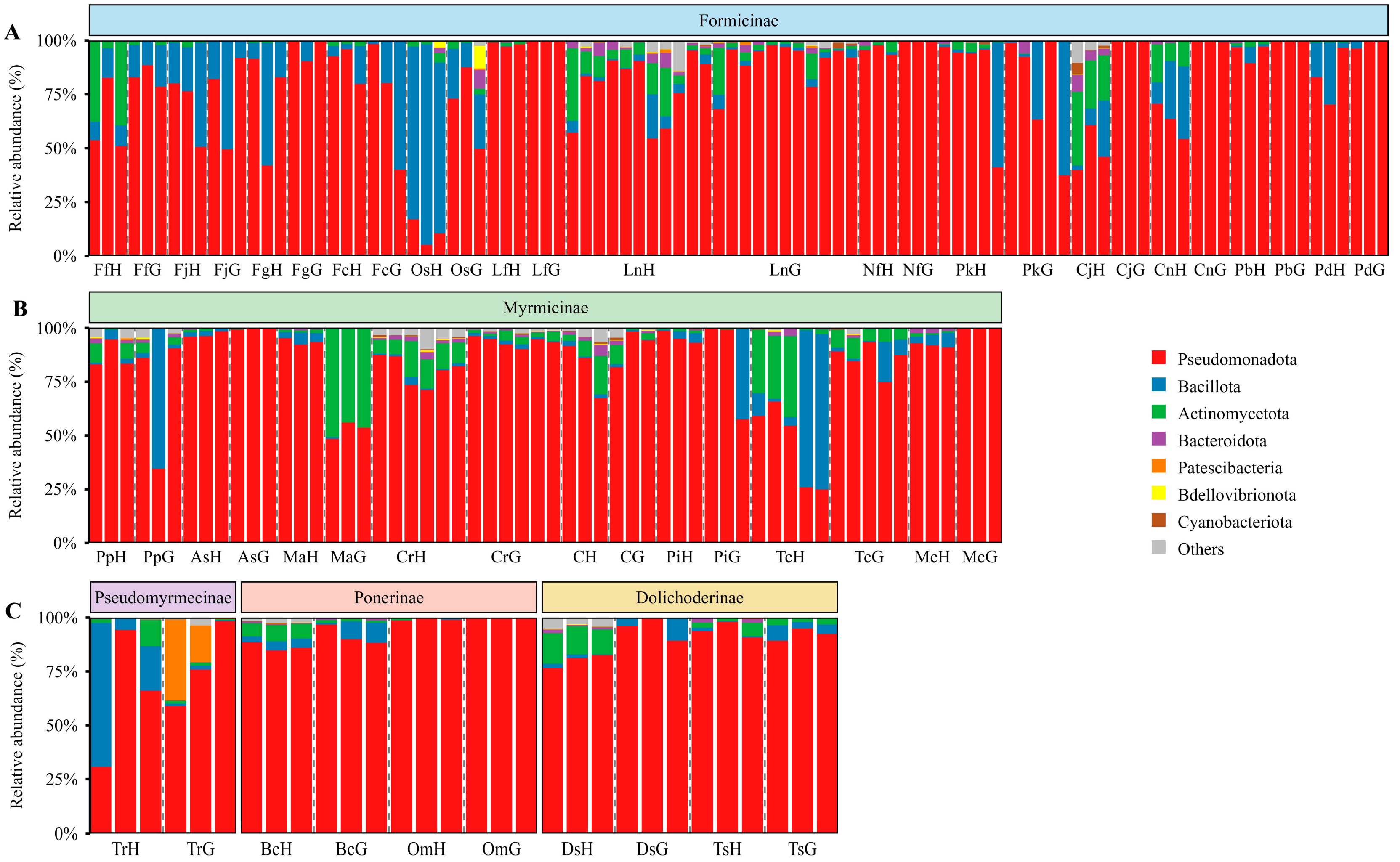

Figure S2. Relative abundance of dominant bacterial phyla in individual head and gaster samples of different ant taxa. (A) Formicinae, (B) Myrmicinae, and (C) Pseudomyrmecinae, Ponerinae, and Dolichoderinae. (Note: Individual bars represent bacterial community profiles from separate colonies. H and G indicate head and gaster samples, respectively. Dashed lines separate different ant species.)

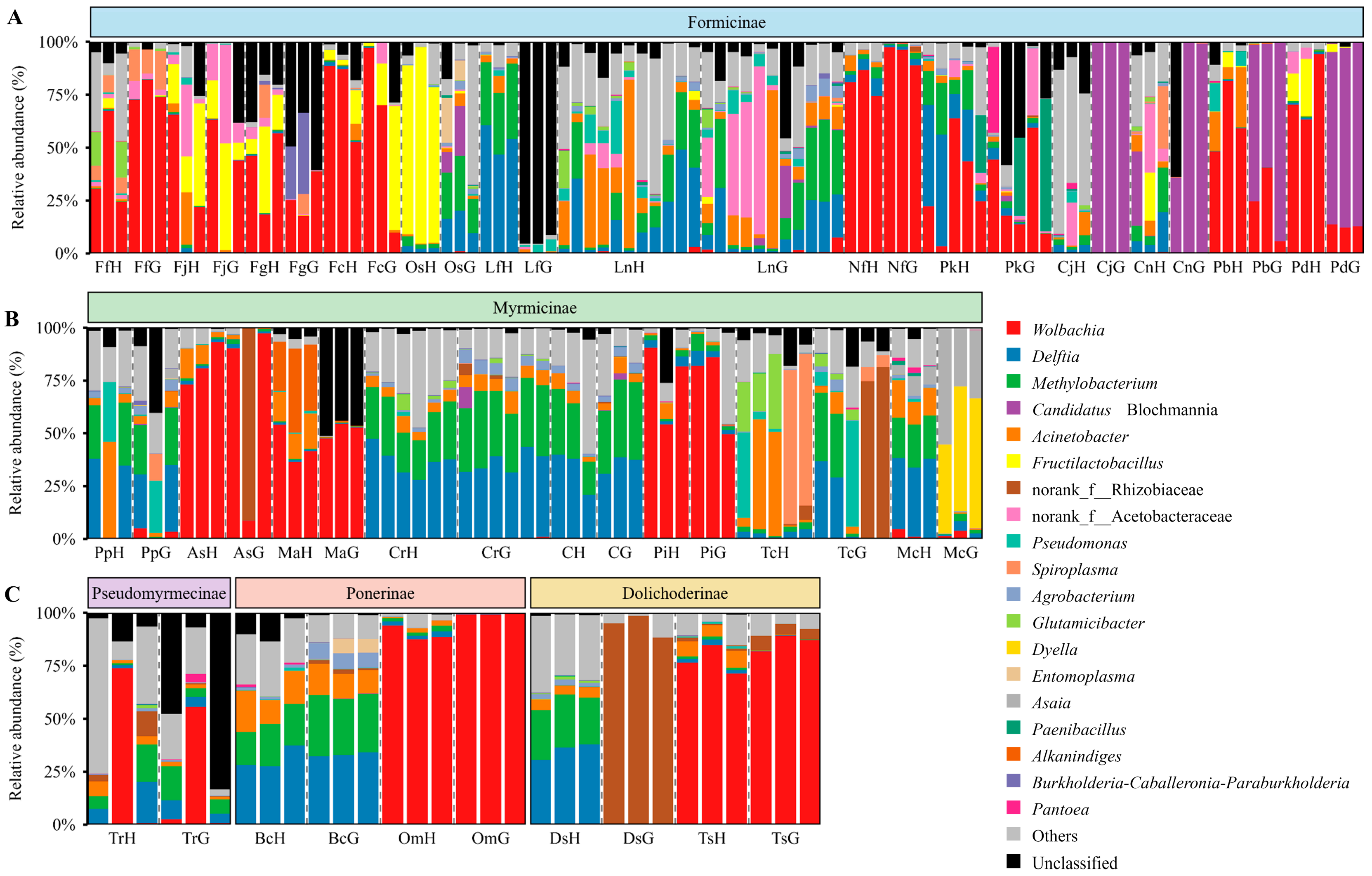

Figure S3. Relative abundance of dominant bacterial genera in individual head and gaster samples of different ant taxa. (A) Formicinae, (B) Myrmicinae, and (C) Pseudomyrmecinae, Ponerinae, and Dolichoderinae. (Note: Individual bars represent bacterial community profiles from separate colonies. H and G indicate head and gaster samples, respectively. Dashed lines separate different ant species.)

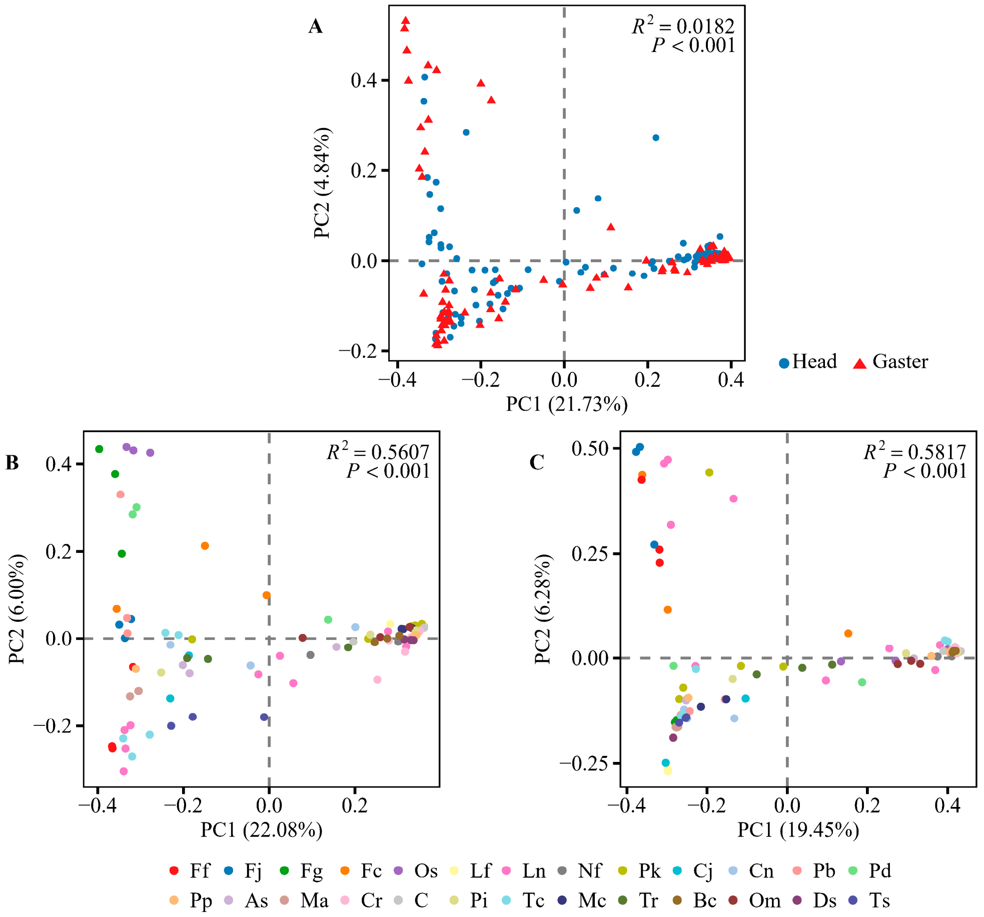

Figure S4. Principal coordinate analysis of bacterial community structure after removal of *Wolbachia* and *Candidatus* Blochmannia. (A) Paired head and gaster samples. (B) Head samples among the 26 sampled ant taxa. (C) Gaster samples among the 26 sampled ant taxa. (Note: Colony Cj2 was excluded because no ASVs remained in sample Cj2G after removal of the two bacterial genera.)
